# Modulation of tumor environment in colorectal cancer – could gut microbiota be a key player?

**DOI:** 10.3389/fgstr.2022.1021050

**Published:** 2022-09-20

**Authors:** Ana Duarte Mendes, Rodrigo Vicente, Marina Vitorino, Michelle Silva, Diogo Alpuim Costa

**Affiliations:** ^1^ Medical Oncology Department, Hospital Professor Doutor Fernando Fonseca, Amadora, Portugal; ^2^ Breast Cancer Unit, CUF Oncologia, Lisbon, Portugal; ^3^ NOVA Medical School, Faculdade de Ciências Médicas, Lisbon, Portugal

**Keywords:** colorectal cancer, microbiota, microbioma, dysbiosis, immunity, immunotherapy, pharmacomicrobiomics

## Abstract

The treatment paradigm of neoplastic diseases has dramatically shifted with the introduction of immune checkpoint inhibitors (ICI). They induce a durable response in a wide variety of solid tumors, but this response depends on the infiltration of lymphocytes capable of recognizing and killing tumor cells. The primary predictor of intrinsic immune resistance to ICIs is the absence of lymphocytes in the tumor, the so-called “cold tumors”. Colorectal cancer (CRC) remains one of the most common and challenging cancer, but it is not traditionally considered a highly immunogenic tumor. In fact, immunotherapy showed a remarkable antitumoral activity only on a small subset of CRC patients – the ones with microsatellite instability-high/deficient DNA mismatch repair (MSI-H/dMMR). Most CRCs display a molecular microsatellite stability/proficient DNA mismatch repair (MSS/pMMR) profile, so strategies to improve tumor immunogenicity are crucial. Therefore, ongoing studies investigate new approaches to convert “cold” to “hot” tumors in MSS/pMMR CRCs. In addition, it has been described that gut microbiota influences tumor development and the host immune response. Hence, the microbiota may modulate the immune response, becoming a promising biomarker to identify patients who will benefit from ICIs. Future data will help to better understand microbiota mechanisms and their role in ICI efficacy. Precision medicine in cancer treatment could involve modulation of the microbiota through different strategies to improve tumor immunogenicity. In this review, we aim to present the potential relationship between gut microbiota and the modulation of the immune system and the hypothetical implications in CRC treatment, namely ICIs.

## Introduction

Colorectal cancer remains one of the most common malignancies. Although overall mortality continues to decline, it remains on the podium of cancer-related death worldwide, with 0.9 million estimated deaths worldwide in 2020 (1, 2). Moreover, notwithstanding the risk of developing CRC increases after the age of 50, it has been increasing dramatically in younger generations, and it is expected to increase by 140% by the year 2030 (3, 4).

The oncogenesis of CRC is multifaceted and encompasses both environmental and genetic factors (5).

The therapeutic approach includes localized therapies, such as endoscopic and surgical excision, radiotherapy, and systemic therapy – chemotherapy, targeted therapy, and immunotherapy, namely immune checkpoint inhibitors (ICI) (6).

ICI has changed the paradigm of cancer therapy by directing the focus to the host instead of the tumor (7). Despite promising results in both hematological and solid tumors, it has failed in most patients with advanced CRC – it only showed significant antitumoral activity in MSI-H/dMMR tumors (8). The current challenge is to overcome this poorly immunogenic profile or, as it has been described, to transform “cold” tumors into “hot” ones (9). One of the most promising areas of immune modulation toward better responses to ICI concerns the inhabitants of our own gut: the gastrointestinal tract is home to trillions of bacteria, most of them commensal. These interact with the host and the immune system, thus constituting a delicate ecosystem called the human gut microbiota (10).

In this review, we will summarize the role of human microbiota in the modulation of the immune system and immunotherapy in CRC.

## Colorectal cancer

The CRC incidence and survival rates have significant disparities between developed and developing countries, making this disease a marker of socioeconomic development. Diagnosis at advanced stages is one of the determinants of these differences (1, 11). In the last ten years, the adoption of screening strategies has contributed to early detection and improved outcome (12, 13). However, statistics globally predict an increase in CRC incidence and exposure to environmental risk factors resulting from a shifting lifestyle (low physical activity, overweight and obesity, excessive consumption of red, processed meats and alcohol, and low dietary fibers) are the main reasons for this evolution (2, 14).

Several critical genes and pathways were identified as crucial factors in the initiation and progression of CRC, such as Wnt, Ras/MAPK, PI3K, TGF-β, P53, and DNA MMR pathways. Classically, investigators biologically divided CRC carcinogenesis mechanisms into two groups: those with MSI and those microsatellite stable but with chromosomal instability (CIN) (11, 15, 16). Two pathological classification systems have been proposed: The Cancer Genome Atlas project and the Consensus Molecular Subtypes. Still, more research is needed to validate their clinical application (17, 18).

The two anatomical locations of the colon have distinct embryonic origins (19, 20). We also found fundamental differences in molecular and clinical characteristics: right colon CRC is usually associated with MSI and the BRAF mutation and is more immunogenic. Left colon CRC is associated with CIN and with mutations in the *APC*, *P53*, and *SMAD4* pathways (19). It remains to be fully understood the biological mechanisms behind such differences.

The MSI tumors are identified in 2–4% of metastatic CRC (mCRC) (20). The subjacent carcinogenesis mechanism depends on the DNA MMR function that ensures the integrity and stability of genetic material by correcting mismatched bases during DNA replication. If any defect occurs in the main MMR proteins MLH1, MSH2, MSH6, and PMS2 or microsatellites, several mutations accumulate, leading to the development of tumors (21). The consequent production of multiple neoantigens induced by genomic mutations is probably one of the mechanisms by which dMMR tumors are sensitive to immunotherapy, even though a complete understanding of the mechanisms leading to improved performance of ICI in dMMR is yet to be attained (22). Furthermore, the inflammatory microenvironment in CRC is an additional feature that makes these tumors more likely to respond to ICI. Hence, evidence has reported the presence of immune cells as CD8+ and CD4+ tumor-infiltrating lymphocytes (TILS), macrophages, and natural killer (NK) cells, as well as an increase in programmed cell death 1(PD-1) and its ligand (PD-L1) in lymphocytes/tumor cells surface (23–25).

Over the last decade, the median overall survival for patients diagnosed with mCRC has doubled (26). Regarding treatment options, fluoropyrimidines alone or combined with oxaliplatin or irinotecan became a standard regimen choice. In resected stage III CRC, fluoropyrimidine alone reduces the risk of death by 10% to 15%, with an additional benefit with an oxaliplatin-based combination (27, 28). Bevacizumab, a humanized monoclonal antibody that inhibits vascular endothelial growth factor (VEGF), and cetuximab and panitumumab, both antibodies targeting the epidermal growth factor receptor (EGFR), are also approved in mCRC according to the right-sided or left-sided colon and RAS gene mutational status. In later lines, TAS-102 improved overall survival (29) and ramucirumab, ziv-aflibercept, and regorafenib are VEGF/VEGF receptor (VEGFR) inhibitors also available in refractory CRC (30, 31).

Regarding the immunotherapy advent, there was an attempt to show the efficacy of ICI in CRC. The results of three phase II studies led to FDA and EMA approval of pembrolizumab and nivolumab (± ipilimumab) for dMMR/MSI CRC previously treated by conventional chemotherapy (31–33). Corroborating this trend, KEYNOTE-177, a phase III trial that compared pembrolizumab with chemotherapy in untreated dMMR mCRC, was responsible for decisive changes in clinical practice, with significant improvement in progression-free survival (PFS) (16.5 months vs. 8.2 months, HR 0.60; 95% CI, 0.45-0.80; *P*=0.0002). Nevertheless, about 30% of patients receiving immunotherapy had disease progression as the best response (34). The phase 2 Atezo-TRIBE trial found that the addition of atezolizumab to chemotherapy and bevacizumab improved PFS in first-line mCRC (35). Later, a significant interaction between MSI status and immunotherapy was observed, with a higher benefit in patients with MSI/dMMR. Little is known about the resistance mechanism to ICIs and tumor heterogeneity in MSI/dMMR tumors. More biomarker-based strategies are needed and a better understanding of the potential synergistic effect of immunotherapy and selective inhibitors of the Ras/BRAF/MEK/ERK pathway to improve patient selection (36).

## Immunotherapy, tumor microenvironment, and patterns of immune response

The ICIs have been used in multiple solid tumors, with good outcomes and prolonged survival confirming their efficacy. Despite the proven clinical benefit, some tumors do not respond to ICI, and this is probably related to specific characteristics of the tumor and the host.

The expression of PD-L1, the constitution of the immune system around the tumor, and the tumor mutational burden (TMB) are fundamental to the success of ICI and are currently considered biomarkers predictive of response (37). The evaluation of the immune profile of patients treated with ICI showed infiltration of immune cells, mainly cytotoxic T cells, in the responders’ group. On the other hand, the lack of immune cells and cytokines led to a resistance to immunotherapy, seen in non-responders’ patients (38).

The tumor bed, designated by the tumor microenvironment (TME), is a complex entity constituted by a heterogeneous collection of cells, secreted factors, and an extracellular matrix. The immune infiltration of the TME can be composed of all types of immune cells (39). Interactions between immune cells and tumor cells influence the environment and produce a pro- or antitumor effect. The TILs are critical cells in the TME, with the majority being T cells (40). Some T cells are related to tumorigeneses, like regulatory T cells (Treg) or helper T cells. In contrast, others are related to the elimination of the tumor, like NK cells and cytotoxic T cells (41).

**Figure 1:**
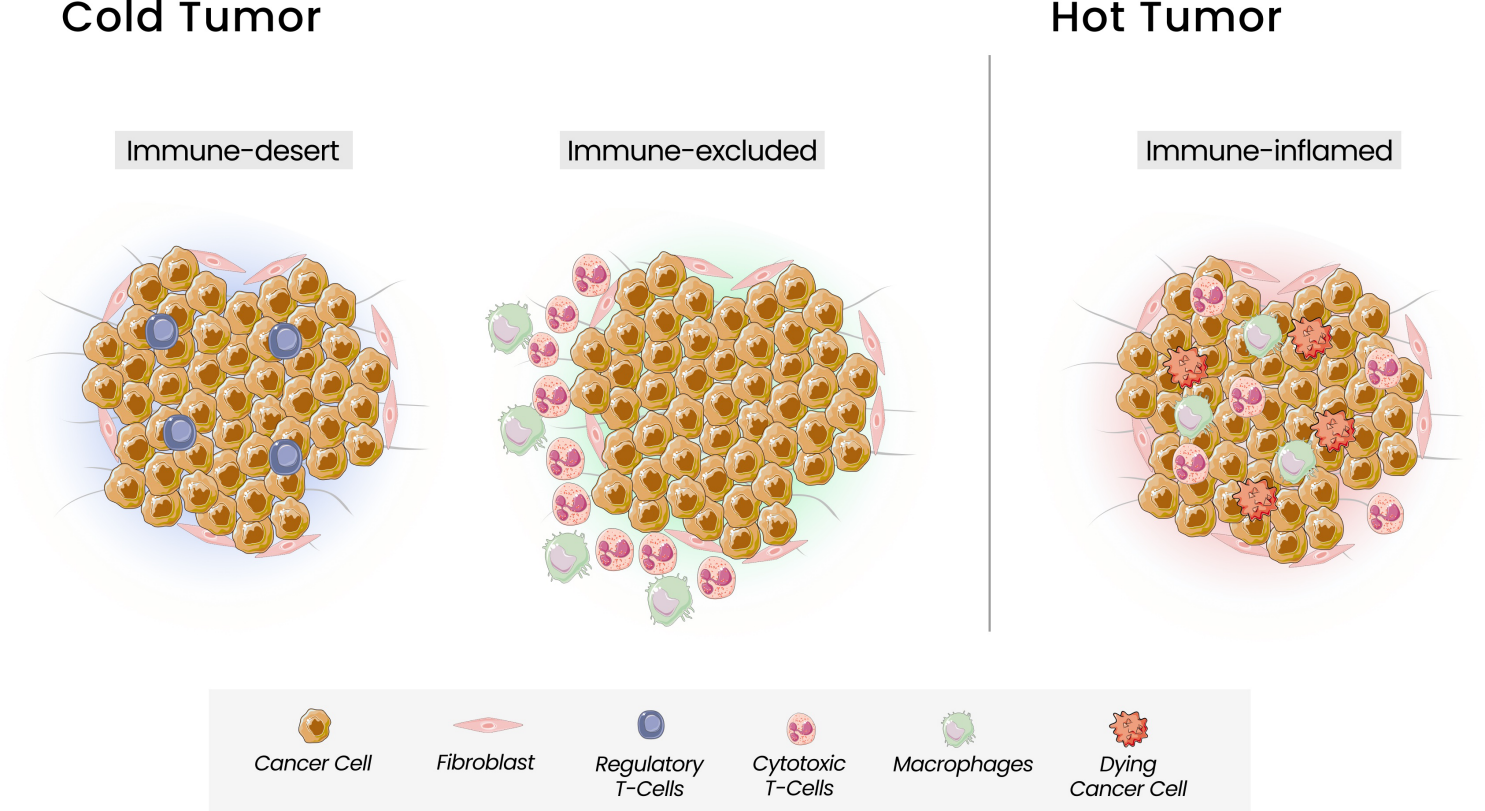
Tumor Immune Phenotypes. Three immunophenotypes are observed according to the spatial distribution of CD8 + T lymphocytes in the tumor microenvironment (TME): the immune-desert, immune-excluded and immune-inflamed phenotypes. In the immune-desert phenotype, immune cells are absent from the tumor and its periphery. In the immune-excluded phenotype, immune cells accumulate but do not efficiently infiltrate. The immune-inflamed is characterized by the infiltration of pro-inflammatory immune cells.

Based on the T cells infiltration, Chen and Mellman defined three types of tumors that can be correlated with response to ICI: the immune inflamed phenotype, or “hot tumor”, associated with a better response, and the cold tumors, the immune-excluded and the immune-desert phenotypes (42) (**Figure 1**). The immune-inflamed phenotype is characterized by the presence of immune cells, such as CD4+, CD8+ T cells, and pro-inflammatory cytokines like interferon (IFN), interleukins 12 and 23, and tumor necrosis factor (TNF)-α. In this phenotype, an antitumor immune response prevails, activating and expanding T cells (39). The immune-excluded and immune-desert phenotypes, considered “cold tumors”, are characterized by a lower response to ICI and a worse prognosis. Despite the presence of immune cells in the immune-excluded phenotype, T cells are located in the stroma surrounding the tumor cells. The tumor, in this case, can promote signaling that blocks dendritic cells and other mechanisms capable of recruiting T cells to the center of the tumor. In the immune-desert phenotype, there is a lack of cytotoxic cells and a prevalence of inhibitory immune cells, like Treg (37). Beyond the paucity of immune effector cells, these last two phenotypes are characterized by a low TMB and a lack of antigen release, reinforcing their poor tumor immunogenicity. The greater the number of mutations in a tumor, the more immunogenic the tumor will be, as these mutations can provide targets for cytotoxic cells. However, some mutations can have the opposite function, acting to attenuate the immune response. Mutations that can decrease the transcription of Major Histocompatibility Complex (MHC) class I molecules will also interfere with peptide loading and presentation process, leading to a weak response (42).

Many steps can inhibit T cells priming and activation in driving immune cells into tumors, leading to a non-inflamed tumor bed. Given these different profiles of tumor behavior, more recent studies try to promote a switch in the tumor environment, turning “cold” into “hot” tumors. Several mechanisms can be used, like the stimulation of recruitment of dendritic cells, stimulation and activation of effector cells, or modification of chemokines and cytokines that can modify the cell traffic and activation (43). Epigenetic modifications, including DNA methylation and chromatin remodeling, can increase tumor immunogenicity and immune recognition, and the subsequent release of pro-inflammatory cytokines. Studies *in vitro* showed that pharmacological or genetic disruption of Treg cells might lead to the acquisition of pro-inflammatory gene signature, with increased CD4+ and CD8+ T cells recruitment to promote antitumor immunity (44). Chemotherapy, radiotherapy, oncolytic viruses, cancer vaccines, or antiangiogenic therapies are currently being studied to improve T cell infiltration. However, it is not enough to increase the number and activity of cytotoxic cells since some components of TME can inhibit their function. One proposed mechanism to convert TME into a “hotter” TME is target therapy against angiogenesis (45). Unfortunately, the clinical benefits are limited since the prolonged use of antiangiogenic therapy increase hypoxia and consequently increase the release of proangiogenic factors (45).

## Human microbiota, immune system, and dysbiosis

The human gut microbiota comprises approximately 3 × 10^13^ bacteria and other highly diverse microorganisms, which are confined to the intestinal lumen. The microbiota is essential in regulating fundamental biological events, and this relationship has evolved into a symbiosis (10, 46, 47). The disruption of this balance, called dysbiosis, is closely related to several diseases, namely infections, autoimmune diseases, cardiovascular diseases, and cancer (48–50). The mutual interaction regulates local and systemic immune homeostasis, maintaining tolerance for commensal bacteria and allows the recognition of potentially pathogenic microorganisms.

The lamina propria beneath the epithelial cells (IECs) harbors immune cells, which encompasses the gut-associated lymphoid tissue (GALT), including antigen-presenting cells such as dendritic cells, T cells, and B cells. The mechanisms through which the microbiota regulates the immune system have been scrutinized over the last few years. Essentially, the various pattern-recognition receptors (PRRs) expressed in IECs and immune cells are thought to recognize microbe-associated molecular patterns (MAMPs) of commensal bacteria (51, 52).

The dendritic cells occupy a prominent role: they are activated by the microbes or by microbe-derived elements (e.g., metabolites, products) *via* interactions with PRRS. When activated, they travel to the mesenteric lymph nodes and orchestrate the differentiation of naïve T cells into effector T cells, mainly Tregs and helper 17 (Th17). A subset of these cells may migrate back to the intestine or enter the systemic circulation, thus locally and systemically modulating the host’s immune system. The Th17 cells mediate the conversion to a pro-inflammatory and antitumor state by secreting immunostimulatory cytokines or directly activating neutrophils, versus Tregs, which release anti-inflammatory cytokines and mediate the conversion to an anti-inflammatory state (51–53). MAMPs or microbe metabolites can also stimulate the immune system through other mechanisms: stimulation of enteric neurons with the release of neurotransmitters that regulate the immune cell function; secretion of immunoglobulin (namely IgA) and their crucial role in the blockade of bacterial adherence, and activation of the innate immune response (53–55).

### Gut microbiota and colorectal cancer

Many of the recognized environmental and lifestyle factors related to CRC are also linked to microbiota dysbiosis (56–58). The gut microbiota is probably at the intersection of these risk factors. As Fearon et al. proposed, the microbiota may be considered an independent driver before the transformation from adenoma to carcinoma (59). The impact of diet on microbiota was thoroughly described by O’Keefe et al., in which a diet exchange between different populations resulted in remarkable changes in microbiota (60). It is also essential to mention the impact of consuming processed foods, as nitrate consumption, rich in processed food, can lead to the formation of N-nitroso compounds by the gut microbiota, some of which are carcinogenic (61, 62).

**Figure 2:**
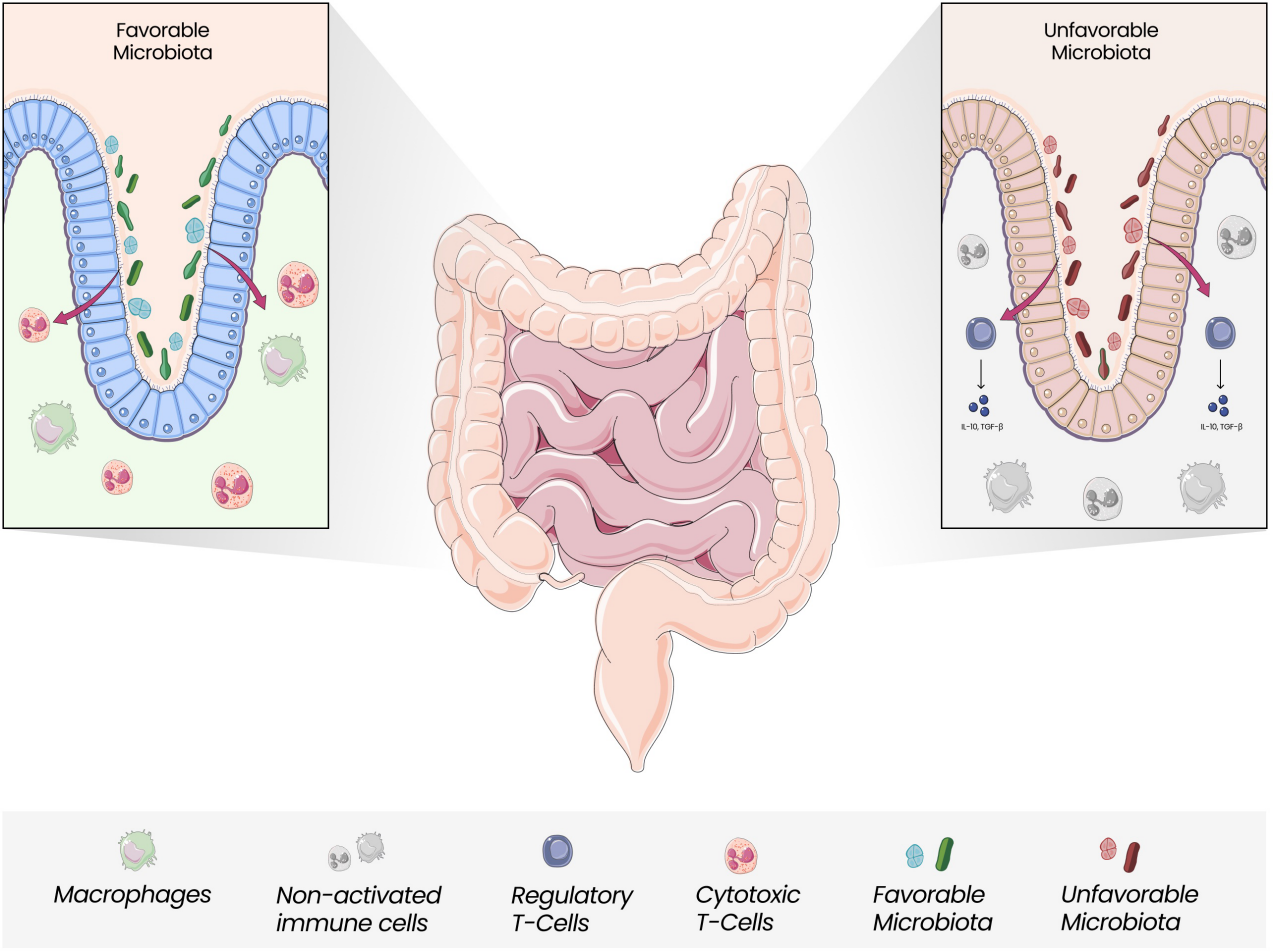
Gut Microbiota and Immune Modulation. Gut Microbiota is closely linked with the modulation of local and systemic immune responses. The unbalanced growth of unfavorable microorganisms, which seems to be more pronounced in the left colon, can mediate less efficient responses regarding antitumor activity, with Tregs releasing anti-inflammatory cytokines and promoting an anti-inflammatory state.

Dysbiosis with the unbalanced growth of certain species, including *Fusobacterium nucleatum, Bacteroides fragilis*, and *Escherichia coli*, along with a reduction in *Roseburia, Clostridia, Clostridium*, and *Clifridia*, can increase the expression of pro-inflammatory cytokines, reduce butyrate-producing bacteria along with enriching pro-inflammatory pathogens and increase the risk of oncogenesis (56, 63–65). (**Figure 2**) Butyrate can induce antitumor responses and help in microbiota homeostasis (66). The overgrowth of *F. nucleatum* has been associated with tumorigenesis through different mechanisms: an increase in M2 macrophages, a decrease in FOXP3+ T cells in the TME, and the presence of bacterial proteins FadA e Fap2, which activate the WNT/β catenin signaling pathway and inhibit NK cells and T cells signaling (67, 68). Other microorganisms are also linked to CRC development: fungal dysbiosis may also induce tumor cell progression (69). Oppositely, *Saccharomyces cerevisiae* could suppress the growth of tumor cells (70). The impact of the microbiota on the biological mechanisms that culminate in the differences between the right and left colon has been questioned. It has been hypothesized that there is an increased amount of pathogenic bacteria in the left colon which could explain the higher incidence of left CRC (71–73).

The microbiota is not only associated with local oncogenesis but has also been proposed as a facilitator of metastasis. Hepatic metastases are preceded by the previous formation of premetastatic niches. This is harbingered by the migration of bacteria to the liver through the portal venous system, and certain bacterial strains, such as *Escherichia coli* C17 or *Proteus mirabilis*, have been strongly associated with this mechanism (74, 75).

Gut microbiota can also improve the effectiveness of the antitumor effect of chemotherapy drugs (76). Other gut microbes might also aggravate chemotherapy-related adverse reactions *via* drugs’microbial metabolism (77).

### Connection between microbiota and immunotherapy

Apart from the relationship with classic CRC chemotherapy, there is also a potential link with targeted agents and ICI. Gut microbiota is a critical modulator of TME, and it might be linked to ICI response in solid tumors. Initial findings by Vetizou et al. showed that the CTLA-4-targeting antibody ipilimumab could treat specific-pathogen-free mice but not germ-free mice (78–81). Multiple gut bacteria were found to be associated with better outcomes in patients treated with anti-PD-1 and anti-CTLA4 immunotherapy (e.g., *Akkermansia muciniphila, Bifidobacterium longem, Faecalibacterium prausnitzii*) *(* 82, 83). It has also been described as a potential influence on immunotherapy-related adverse events (84).

Even though the mechanisms by which the gut microbiota influences immunotherapy remain under study, research appears to focus on three themes: bacteria or bacterial components that stimulate antitumor T-cell responses, molecular mimicry between bacteria and tumor epitopes, and bacterial metabolites that shape antitumor immunity (85–87). The interpretation of data linking ICI and the microbiota can be hampered by several factors: small cohorts, variable definitions of response, and the confounding factors linked to gut microbiota composition (diet, treatment, geography, ethnicity, etc.).

Given the apparent benefits in the presence of certain bacteria species, one may ask whether it will be possible to modulate the microbiota with the final aim of attuning the immune system. Microbiota modulation has been receiving widespread attention (**Table 1**). It can occur directly through actions of dietary components on the microbiota’s composition or metabolic processes or indirectly through altering the gut physiology to change the intestinal lumen environment, thereby producing changes in the microbiota. So far, the main ways of modulating the microbiota are through diet, administration of prebiotics and probiotics, and fecal microbiota transplantation. Concerning dietary habits, data have shown a profound and beneficial metamorphosis in the microbiota composition with a high-fiber, low-fat diet (60). The administration of growth substrate (prebiotics) to induce the growth of specific strains has also shown potential in modulating the microbiota (99).

**Table 1:** Clinical studies regarding modulation of gut microbiota and cancer treatment.

Concluded Clinical Studies				
	Reference	Study Population	Intervention	Results
Analysis of Fusobacterium persistence and antibiotic response in colorectal cancer (88)	Bullman et al. Science 2017	CRC	Treatment with metronidazole	Significant decrease in *Fusobacterium* load in the tumor tissue (P = 0.002) as well as a significant reduction in tumor cell proliferation (P = 0.002).
Phage-guided modulation of the gut microbiota of mouse models of colorectal cancer augments their responses to chemotherapy (89)	Zheng et al. Nat Biomed Eng. 2019	CRC	Irinotecan-loaded nanoparticles linked to phages	Decrease in the numbers of Fusobacterium nucleatum (P = 0.01); median survival in mice increased from 20d to 42d.
Aspirin Modulation of the Colorectal Cancer-Associated Microbe Fusobacterium nucleatum (90)	Brennan et al. mBio. 2021	CRC	Administration of aspirin	Decrease in fusobacterial abundance in colon adenoma tissue.
A randomized double-blind trial on perioperative administration of probiotics in colorectal cancer patients (91)	Gianotti et al. World J Gastroenterol. 2010	CRC	Administration of probiotics perioperatively	*Lactobacillus johnsonii reduces* the concentration of pathogens and modulates local immunity.
Intestinal microbiota is altered in patients with colon cancer and modified by probiotic intervention (92)	Hibberd et al. BMJ Open Gastroenterol. 2017	CRC	Administration of probiotics	Increased abundance of butyrate-producing bacteria, especially Faecalibacterium and Clostridiales spp. CRC-associated genera such as Fusobacterium and Peptostreptococcus tended to be reduced in the fecal microbiota of patients that received probiotics.
Effects of prebiotics on immunologic indicators and intestinal microbiota structure in perioperative colorectal cancer patients (93)	Xie et al. Nutrition 2019	CRC	Administration of probiotics	Preoperative period: increased serum levels of IgG; P = 0.02), IgM (P = 0.00), and transferrin (P = 0.027; all P < 0.05). Postoperative period: enhanced levels of IgG (P = 0.003), IgA (P = 0.007), suppressor/cytotoxic T cells (CD3^+^CD8^+^; P = 0.043), and total B lymphocytes (CD19^+^; P = 0.012) Prebiotics increased the abundance of Bifidobacterium (P = 0.017) and Enterococcus (P = 0.02; both P < 0.05) but decreased the abundance of Bacteroides (P = 0.04)
Impact of the preoperative use of synbiotics in colorectal cancer patients: A prospective, randomized, double-blind, placebo-controlled study (94)	Polakowski et al. Nutrition 2018	CRC	Administration of synbiotics	Significant reductions in IL-6 levels (163.2 ± 19.5 versus 138.8 ± 12.5, P < 0.001) and CRP (10 ± 5.2 versus 7.17 ± 3.2, P < 0.001).
**Ongoing Interventional Trials**				
	**NCT Trial No.**	**Phase**	**Study population**	**Intervention**
Feasibility Study of Microbial Ecosystem Therapeutics (MET-4) to Evaluate Effects of Fecal Microbiome in Patients on ImmunOtherapy (MET4-IO) (95)	NCT03686202	Phase I	Solid Tumors	Microbial Ecosystem Therapeutics (MET-4) in patients on immunotherapy
A Phase I/II Open Label, Safety And Preliminary Efficacy Study of MRx0518 In Combination With Pembrolizumab In Patients With Advanced Malignancies Who Have Progressed On PD-1/PD-L1 Inhibitors (96)	NCT03637803	Phase I/II	Solid Tumors	MRx0518 in combination with pembrolizumab
Phase II, Single-arm Study of FMT Combined With Immune Checkpoint Inhibitor and TKI in the Treatment of Colorectal Cancer Patients With Advanced Stage (97)	NCT05279677	Phase II	CRC	Fecal microbiota transplantation in combination with Sintilimab and Fruquintinib
Preoperative Endoscopic Treatment With Fosfomycin and Metronidazole in Patients With Right-sided Colon Cancer and Colon Adenoma: a Clinical Proof-of-concept Intervention Study MEFO Trial (98)	NCT04312360	Phase II	CRC and Colon Adenoma	Therapeutic endoscopy with metronidazole and fosfomycin disodium

CRC, colorectal cancer; CRP, C-reactive protein; FMT, fecal microbiota transplant; IL, interleukin; Ig, immunoglobulin; PD-1, programmed cell death protein 1; PD-L1, programmed death-ligand 1; TKI, tyrosine kinase inhibitor.

The direct introduction of bacteria, either in the form of a fecal transplant or of just a few microorganisms (specific strain or consortium) with probiotics has an undeniable role in the microbiota regulation to improve the host immunity (100, 101). Fecal microbiota transplantation is being experimentally used to treat metabolic diseases, inflammatory bowel disease, and cancer (102–106). Conversely, eradicating specific microorganisms with certain antibiotics, such as metronidazole, is also an active field of investigation (88).

Interestingly, vitamins appear to modulate microbiota as well: vitamin D is linked with anti-inflammatory and immune-modulating properties in the gut (107). Promising new research on colon-delivered vitamin B3 is associated with improving biomarkers for inflammation (108).

## Conclusion

A complex tie lies between the host and gut microbiota.

In human diseases, gut microbiota mediates the immune response, modulating disease development and progression, and potentially interfering with treatment efficacy. It may play a key role also in human cancer, including the ability to modulate host immune response (109, 110). The gut microbiota may influence the anti-tumor activities by producing specific metabolites or inducing T-cell responses. On the contrary, some bacterial species improve tumor proliferation and metastasis, and understanding those interactions in the context of cancer is crucial in the quest for potential therapeutic targets. In this context, there is a shred of increasing evidence for the correlation of gut microbiota with cancer immunotherapy activity and toxicity (111).

The modulatory effect of the gut microbiota on ICI response may create new therapeutic opportunities. In MSI-H patients with intrinsic/*de novo* and acquired resistance settings, it may become essential to examining the microbiota.

Despite the advances, the underlying mechanisms, the therapeutic impact, and which specific microbes and immune cells interact with each other remain obscure. Moving forward, clinical trials will undoubtedly spur efforts to examine the influence of the immune-gut interaction on immunotherapy treatment in clinical settings.

Hopefully, this will quickly become much more than just a gut feeling.

## Author contributions

ADM and DAC contributed to the conception and design of the review. ADM, RV and MV wrote the first draft of the manuscript. MS and DAC revised the manuscript. All authors read and approved the submitted version.

## Conflict of interest

The authors declare that the research was conducted in the absence of any commercial or financial relationships that could be construed as a potential conflict of interest.

## Publisher’s note

All claims expressed in this article are solely those of the authors and do not necessarily represent those of their affiliated organizations, or those of the publisher, the editors and the reviewers. Any product that may be evaluated in this article, or claim that may be made by its manufacturer, is not guaranteed or endorsed by the publisher.
